# Gender Difference on the Association between Dietary Patterns and Obesity in Chinese Middle-Aged and Elderly Populations

**DOI:** 10.3390/nu8080448

**Published:** 2016-07-23

**Authors:** Ya-Qun Yuan, Fan Li, Pai Meng, Jie You, Min Wu, Shu-Guang Li, Bo Chen

**Affiliations:** Key Laboratory of Public Health Safety of Ministry of Education, Collaborative Innovation Center of Social Risks Governance in Health, School of Public Health, Fudan University, Shanghai 200032, China; 14211020024@fudan.edu.cn (Y.-Q.Y.); 11307120186@fudan.edu.cn (F.L.); 12211020059@fudan.edu.cn (P.M.); 13211020024@fudan.edu.cn (J.Y.); wumin@shmu.edu.cn (M.W.); leeshuguang@fudan.edu.cn (S.-G.L.)

**Keywords:** dietary patterns, obesity, gender difference, factor analysis, middle-aged and elderly Chinese people

## Abstract

Dietary patterns are linked to obesity, but the gender difference in the association between dietary patterns and obesity remains unclear. We explored this gender difference in a middle-aged and elderly populations in Shanghai. Residents (*n* = 2046; aged ≥45 years; 968 men and 1078 women) who participated in the Shanghai Food Consumption Survey were studied. Factor analysis of data from four periods of 24-h dietary recalls (across 2012–2014) identified dietary patterns. Height, body weight, and waist circumference were measured to calculate the body mass index. A log binominal model examined the association between dietary patterns and obesity, stratified by gender. Four dietary patterns were identified for both genders: rice staple, wheat staple, snacks, and prudent patterns. The rice staple pattern was associated positively with abdominal obesity in men (prevalence ratio (PR) = 1.358; 95% confidence interval (CI) 1.132–1.639; *p* = 0.001), but was associated negatively with general obesity in women (PR = 0.745; 95% CI: 0.673–0.807; *p* = 0.031). Men in the highest quartile of the wheat staple pattern had significantly greater risk of central obesity (PR = 1.331; 95% CI: 1.094–1.627; *p* = 0.005). There may be gender differences in the association between dietary patterns and obesity in middle-aged and elderly populations in Shanghai, China.

## 1. Introduction

Obesity is defined as abnormal or excessive fat accumulation, which is a major risk factor for various metabolic disorders, such as hypertension, impaired glucose tolerance, dyslipidemia, and pro-inflammatory status. Obesity and obesity-related diseases are increasing dramatically and have become a massive burden on the world economy and global health. Worldwide, the prevalence of being overweight increased from 24.6% in 1980 to 34.4% in 2008, and the prevalence of obesity nearly doubled from 6.4% to 12.0% during the same 28-year period [1]. On the basis of World Health Organization (WHO) statistics, 39% of adults aged 18 years and over were overweight in 2014, and 13% were obese [2]. In China, the prevalence of overweight and obesity were 16.4% and 3.6% in 1992 [3], and 22.8% and 7.1% in 2002 [4], respectively. According to the China National Diabetes and Metabolic Disorders Study, 31.4% and 12.2% (approximately 299.5 and 116.2 million, respectively) of Chinese adults were overweight and obese in 2012 [5]. As a consequence, overweight and obesity have been estimated to cause 3.4 million deaths, with 4% of years of life lost and 4% of disability-adjusted life-years worldwide in 2010 [6]. 

China has entered into an aging society and will continue to age rapidly in the future. By the end of 2013, the population aged 60 and over accounted for 14% of the total population. It is predicted that 25% of the population will be aged 60 years and over by 2035 [7]. As the first city to enter into an aging society in China, Shanghai, had an aging population 20 years earlier than the rest of the country. In Shanghai, the proportion of the population aged 60 years and over increased from 10.7% in 1979 to 25.7% in 2012, and by the end of 2015, the ratio reached 30% [8]. The emergence of an aging population in China will significantly increase the prevalence of chronic non-communicable diseases, including diabetes, cancers, chronic respiratory diseases, and cardiovascular diseases [9]. The middle-aged population (aged 45–60 years) is more sensitive to the health risk factors and more likely to be influenced by the non-communicable chronic diseases because of the apparent hormonal changes they experience. Therefore, obesity in the middle-aged and elderly population has been a serious concern for China, and the mechanism by which it develops requires deeper exploration.

Obesity is a complicated multifactorial chronic disease, which may be caused by the interaction between genetic predisposition, the environment and human behavior [10]. Dietary structure has been demonstrated as a determinant factor to obesity; however, the association is inconsistent in different populations and is poorly understood [11]. Compared with traditional dietary analysis, which simply focused on the relationship between individual nutrients or foods, the analysis of dietary patterns has emerged as a holistic and comprehensive approach [12]. A dietary pattern can take advantage of the intricate dietary data, and take account of total dietary consumption and the potential interaction between many nutrients and foods [13,14,15]. These advantages have led to the analysis of dietary patterns being used widely to determine the association between diet and related chronic diseases in nutritional epidemiology in recent decades [16,17,18,19,20].

Dietary patterns vary according to age, ethnicity, culture and other lifestyle factors [21]. Previous studies have reported an association of dietary patterns with obesity in Chinese middle-aged and elderly people [22,23]. A recent study in Japan reported a gender difference in the relationship between dietary patterns and type 2 diabetes [20]. However, to the best of our knowledge, no study has explored the effect of gender difference on the associations between dietary patterns and obesity in Chinese middle-aged and elderly people. Therefore, this study identified the major dietary patterns among a Chinese population aged 45 years and over, and examined the effect of gender difference on the associations of these patterns with obesity.

## 2. Study Population

**Figure 1:**
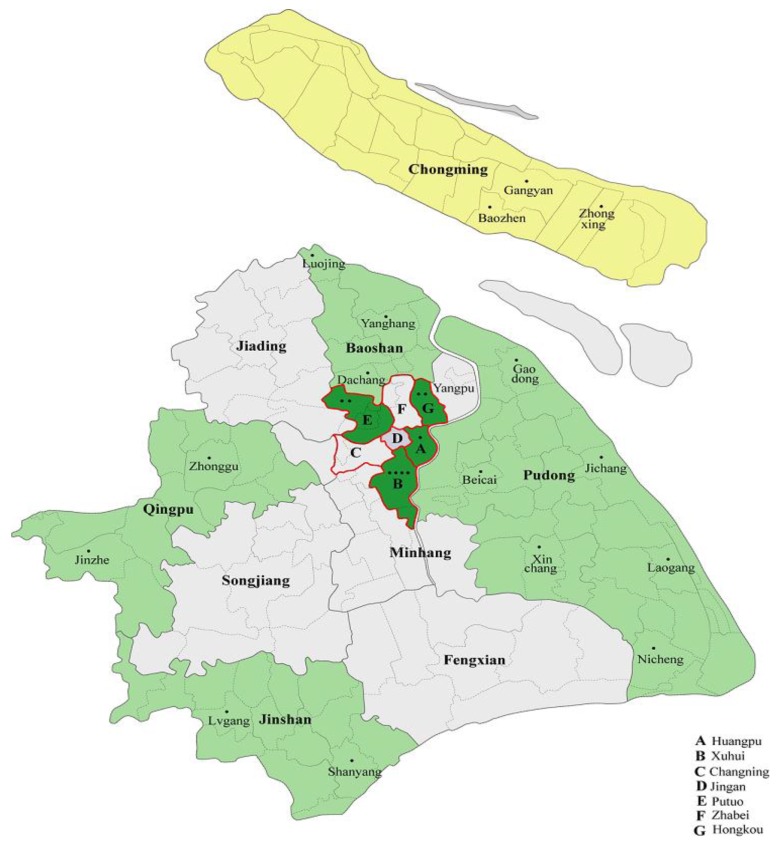
Multi-stage cluster random sampling of the Shanghai Food Consumption Survey.

The Shanghai Food Consumption Survey (SHFCS) was launched during September 2012 through August 2014. This cross-sectional study was designed to acquire knowledge of the dietary structure and nutritional status of adults in Shanghai, China. A multi-stage cluster random sampling method was used to draw the study sample from nine of 18 districts or counties in Shanghai, including the eight districts of Huangpu, Xuhui, Putuo, Hongkou, Jinshan, Pudong, Qingpu, and Baoshan, and the county of Chongming (Figure 1). One to six residential communities were selected randomly from each district or county, according to the population density. The project was conducted four times across two years (autumn 2012, spring and winter 2013, and summer 2014). A total of 2291 participants aged over 45 years attended at least one survey session. After exclusion of 106 participants because of their incomplete anthropometric information, as well as 139 participants who had missing information on their food consumption (29) or health related factors (110), 2046 participants aged 45 years and over were ultimately enrolled. Among them 690 participants completed all four surveys. The present analysis was based on the SHFCS, which included 968 men and 1078 women aged 45 years and over with completely demographic, anthropometric, and dietary data. All subjects submitted written informed consent before their participation in the survey. The study was approved by the local authorities and the Ethics Committee of the School of Public Health at Fudan University.

## 3. Dietary Assessment

Dietary intake was measured four times using one-day 24-h dietary recalls (once a season) during the SHFCS. Valid forms were used by experienced interviewers to administer the 24-h dietary recalls via a household interview. Each participant was asked to report the types and amount of all foods (measured in g) that they consumed both at home and away from home during the previous 24 h. The average intake of the four recalls was used for each individual. A total of 408 kinds of food items were collected in the survey. The 408 varieties of food items were divided into 25 food groups, based on a combination of nutritional characteristics of each food mentioned in the Chinese Food Composition 2002 [24] and recommendations about food classification in related studies [11,25]. The 25 food groups were rice; wheat; deep-fried wheat; instant noodles; coarse grains; starchy roots and tubers; vegetables; fruits; pork; poultry; other livestock meats; organ meats; processed meats; fresh water fish and seafood; dairy; legumes; eggs; seeds and nuts; fungi and algae; western fast food; cakes and pastries; candy and chocolates; soft drinks; alcoholic beverages; and tea. The typical foods in each food group are represented in Table 1.

## 4. Anthropometric Measurements

Trained investigators measured anthropometric variables based on a standard protocol. Height was measured to the nearest 0.1 cm with subjects standing without shoes. Weight in relatively light clothes was measured to the nearest 0.1 kg using a calibrated digital scale. Waist circumference (WC) was measured half way between the lower rib edge and the upper iliac crest using a metric measure with an accuracy of 0.1 cm. Body mass index (BMI) values were divided into four categorical levels according to the recommendation of the Working Group on Obesity in China, which are underweight, BMI < 18.5 kg/m^2^; normal, BMI 18.5–23.9 kg/m^2^; overweight, BMI 24.0–27.9 kg/m^2^, and general obesity, BMI ≥ 28 kg/m^2^ [26]. Abdominal obesity was defined as WC ≥ 85 cm for men and ≥80 cm for women in a Chinese population [27].

## 5. Other Health Related Variables

Other health-related variables were collected by skilled interviewers using a general questionnaire. Education level was divided into binary variables (lower and higher) from five initial education categories: illiteracy, primary school, junior middle school, high middle school, bachelor’s degree and above. Marital status was allocated as married or other marital status. Occupation was categorized as retired or other. Information on smoking status included three categories of never smokers, former smokers, and current smokers (at least one cigarette per day now). Physical activity was identified as metabolic equivalents in hours per week (MET-h/week), and was divided into three levels: light, moderate, and vigorous. Housework status was allocated to two categories (Yes/No), based on the question “did you do the house work in your daily life?” Total energy intake was calculated according to the China Food Composition 2002 [26], expressed in kilocalories per day (kcal/day).

## 6. Statistical Analysis

Exploratory factor analysis (principle components) was used to identify dietary patterns on the basis of the 25 food groups mentioned above. Data adequacy for factor analysis has been assessed by the Kaiser-Meyer-Olkin Measure of Sample Adequacy and the Bartlett Test of Sphericity. Factors were rotated by orthogonal transformation (varimax rotation) to minimize the correlation among them and to improve their interpretability. After evaluating the Eigen values, scree plot, factor interpretability, and variance explained, four factor solutions were selected. Food groups were considered to contribute to the pattern significantly if they had an absolute correlation (factor loading) ≥0.20 with that pattern. The factor scores for every dietary pattern were calculated for each participant by summing intakes of food groups weighted by their factor loadings. The labeling of dietary patterns was done on the basis of interpretation of major food groups with high factor loadings in each dietary pattern. Participants were categorized into quartiles according to factor scores of each pattern. Quartile 1 represent a lower consumption of this food pattern, while quartile 4 represented a higher one. Data are expressed as the mean ± standard deviation (SD) for continuous variables and as percentages for categorical variables. A chi-squared test for categorical variables and analysis of variance (ANOVA) for continuous variables were used to evaluate the association between health-related factors and dietary patterns. A log binomial model was used to estimate the prevalence ratios (PR) and 95% confidence interval (95% CI) for general obesity and abdominal obesity across the quartile categories of dietary pattern scores. Model 1 was adjusted for age (continuous). Model 2 was additionally adjusted for education level (lower, higher), marital status (married, others), smoking status (never, former, current, but not included in women), occupation (retired, others), physical activity (light, moderate, and vigorous), and housework status (yes/no). Model 3 was further adjusted for total energy intake (continuous). The statistical analysis was performed using SPSS software package version 22.0 for windows (SPSS Inc., Chicago, IL, USA) and SAS software version 9.3 for Windows (SAS Institute Inc., Cary, NC, USA). Two-side *p*-values < 0.05 were considered statistically significant.

## 7. Results

Selected individual characteristics of the study sample are displayed in Table 2, stratified by gender. Men made up 47.3% of the sample and had a similar age distribution to the women. The prevalence of overweight and general obesity were 47.8% for men and 43.9% for women; likewise, compared with women (42.7%), men had a higher prevalence of abdominal obesity (52.9%). Most participants in both genders were married (91.1%). Men (33.3%) reported a higher education level than women (27.1%); however, more women were retired (66.4% vs. 59.1%) and did more housework (92.5% vs. 67.9%) compared with men. In terms of smoking status, the majority of male participants were never smokers (42.9%) and current smokers (51.8), while almost all of the female participants were non-smokers. No significant difference was found in the level of physical activity between men and women.

Factor analysis revealed four independent dietary patterns, which explained 26.85% of the variance in total food consumption for men, and 26.22% for women (Table 3). Both genders shared comparable, but not completely consistent, dietary patterns. The rice staple pattern was loaded heavily on rice, vegetables, pork, poultry, fungi, and algae for both genders; however, men ate more starchy roots/tubers, organ meats, processed meats, and seeds/nuts, and women ate more seafood and eggs. The wheat staple pattern was characterized by a high intake of wheat, deep-fried wheat, dairy, instant noodles, legumes and tea, with less rice and starchy roots/tubers for men. In women, this pattern was characterized by a high intake of wheat, coarse grain, fruits, dairy, legumes, seeds/nuts, fungi/algae, soft drinks, and tea, and less rice and processed meats. The snacks pattern was characterized by high consumption of starchy roots/tubers, fruits, cakes/pastries, soft drinks, alcoholic beverages and tea, but less rice, vegetables, pork and eggs for men; in women, this pattern was represented by high intake of starchy roots/tubers, fruits, seed/nuts and cakes/pastries, but less intake of pork. The last factor was labeled the prudent pattern because of its high intake of coarse grains, fruits, dairy, and tea for men; and coarse grains, fruits, processed meats, and seafood for women. The absolute amount of the 25 food groups across quartiles of dietary pattern scores are displayed in Table A1, which matches with the results of factor analysis in Table 3.

Characteristics of the participants in our study across the quartile categories of dietary scores are shown in Table 4. Subjects of both genders in the top quartile of the rice staple pattern were more likely to be significantly younger and married. Furthermore, men with higher scores for this pattern are more likely to be working and had a higher prevalence of general and abdominal obesity, while women following this pattern are more likely to be a house worker and have a lower prevalence of abdominal obesity compared with those in the lowest quartile. Both genders in the highest quartile of the wheat staple pattern were more likely to have higher education level, to be working and to take more physical exercise; however, men had a higher prevalence of general obesity. Compared with those in the lowest quartile, men and women with higher scores for the snacks pattern were more likely to be older, working, and have a higher level of education. Meanwhile, men in the top quartile of this pattern were more likely to participate in more physical exercise, and women were less likely to be married. In addition, both genders in the top quartile of the prudent pattern were more likely to have a higher level of education, be working, and to participate in more physical exercise. In particular, men with high scores for this pattern were likely to be older, smoke less, and do housework. 

The relationship between general and abdominal obesity and dietary patterns, as assessed by log binomial model is displayed in Table 5. After adjustments for all confounders (age, education level, married status, occupation, smoking status, physical exercise, housework status and energy intake), men in the highest quartile of the rice staple pattern had a significant risk of abdominal obesity (PR = 1.358; 95% CI: 1.132–1.639; *p* = 0.001). By contrast, women with higher scores for this pattern were less likely to have general obesity (PR = 0.745; 95% CI: 0.673–0.807; *p* = 0.031). Meanwhile, the wheat staple pattern for men was also positively associated with abdominal obesity (PR = 1.331; 95% CI: 1.094–1.627; *p* = 0.005). Nevertheless, the snacks and prudent patterns showed no association with the risk of general and abdominal obesity in either gender.

## 8. Discussion

The present study extracted four distinct dietary patterns for a middle-aged and elderly population in Shanghai: the rice staple pattern, wheat staple pattern, snacks, and prudent pattern. Our findings revealed that men who followed a rice staple pattern had a greater risk of general and abdominal obesity. Meanwhile, the same pattern showed a protective effect against general obesity in women. Additionally, the wheat staple pattern was found to have a positive association with abdominal obesity only in men. The effect of gender difference on the relationship between diet and relative health outcomes has been analyzed. Studies in Japan [20] and South Korea [28] reported the effects of gender difference on the association between dietary patterns and type 2 diabetes and metabolic syndrome, respectively. To the best of our knowledge, this is the first study to analyze the effect of gender difference on the relationship between dietary patterns and obesity in middle-aged and elderly Chinese.

The rice staple pattern represents a classical, comprehensive diet structure in South China. The pattern for men was found to have a positive association with general and abdominal obesity. This result was consistent with a study from Zhejiang province China [22], which reported a similar animal food pattern, mainly comprising rice, mushroom, red meat, and fat/oils. Compared with men, women who followed this pattern had a decreased risk of general obesity, which was comparable to a study in Chinese young women that reported a diet resembling the traditional southern pattern (high intake of rice, tubers, vegetables, and pork), which was associated positively with a decreased risk of general and abdominal obesity. Although both genders shared the same label, discrepancies in food composition of this pattern between men and women was the primary cause that led to a significant gender difference on the association between the pattern and obesity. Proteins providing food in the rice staple pattern for men were pork, poultry, organ meats, processed meats, and were pork, poultry, seafood, and eggs for women. Obviously, high consumption of various livestock and poultry meats is the most important factor contributing to obesity. Known as an energy-dense food that contains abundant saturated fat and cholesterol, the association between excess meat consumption and obesity has been confirmed through studies across different populations. The EPIC-PANACEA study found that excess consumption of red meat, poultry, and processed meat might contribute to an increased risk of obesity [29]. In addition, a systematic review and meta-analysis reviewed that red and processed meat intake is directly associated with the risk of obesity and higher BMI and WC [30]. Furthermore, a study in a Chinese population also concluded that greater intake of fatty fresh red meat is significantly associated with a higher WC (men only) and risk of abdominal obesity [31]. Instead of organ and processed meat, women eat more fish and eggs. Fish was indicated to have a protective role against obesity because of its rich content of omega-3 polyunsaturated fatty acid (omega-3PUFA) [32]. Eggs are lower in fat than meat, and those present are mostly the healthy unsaturated fats. Rice is one of the most essential staple foods in China, especially in South China. In general, rice is considered to have a protective effect against obesity because of its lower energy density. However, the relationship between rice and obesity is inconsonant in various populations. Two studies from Jiangsu [33] and Zhejiang province [22] in China both indicated that intake of rice was inversely associated with being overweight. However, this was inconsistent with a study in Korean adults, which showed that the rice-kimchi pattern, characterized by a higher intake of white rice, kimchi, and vegetables, was positively associated with obesity [34]. In our study, rice showed a protective role in the rice staple pattern only for women but not for men. These discrepancies could be attributed to the differences in the population, rice varieties, cooking methods and study design. However, the present study suggests that the comprehensive influence of other foods remaining in a pattern could be another logical explanation. Another food item that showed a different effect between men and women, but in the same pattern are vegetables. Vegetables are considered healthy constituents in the diet, and prevent weight gain through their low energy density and high dietary fiber content. However, in this study, vegetables showed a protective effect only for women, but not for men, which could be attributed to the high smoking prevalence in men. A study of Europeans reported that for persons who stop smoking, high vegetable intakes might be recommended to reduce the risk of overweight [35]. A similar study also concluded that in a large population, higher baseline vegetable intakes did not substantially influence midterm weight change overall but could help to reduce risk of weight gain in persons who stop smoking [36]. In our study, the prevalence of current smoker for men is 51.8%, but very few women smoked. Therefore, smoking might reduce the beneficial impact of vegetables on obesity in men.

The wheat staple pattern was the second dietary pattern in our study, which consisted of different food items, and showed inconsistent associations with obesity between men and women. Men with this pattern consumed high levels of wheat flour, deep fried wheat, instant noodles, dairy, legumes, and tea, and were discovered to have an increased risk of abdominal obesity. One primary characteristic of this pattern for men is high carbohydrate intake. Studies based on other populations suggested that a high carbohydrate diet had positive association with general and abdominal obesity [37,38]. A potential explanation for the mechanism underlying the increased carbohydrate-related risk for obesity is that carbohydrate intake might alter lipid profiles, such as an increase in triglycerides and/or a decrease in high-density lipoprotein cholesterol, leading to increased obesity [39]. In addition, an animal experiment study reported that wheat gluten promotes weight gain in animals on both a high-fat and control-standard diet, partly by reducing the thermogenic capacity of adipose tissues. Moreover, compared with rice, wheat flour absorbs less water when cooked, so the energy density of wheat may be higher than rice. Another characteristic of the wheat staple pattern for men is the unhealthy cooking method, deep-frying. Typical Chinese fast foods, such as deep-fried wheat foods (deep-fried dough sticks and deep-fried dough cakes) and instant noodles, are very popular in China for their tastiness and convenience. However, the deep-fried cooking method of these foods has been a serious concern. A study of three US cohorts suggested that consumption of fried food could interact with genetic background in relation to obesity [40]. The SUN project in a Mediterranean cohort concluded that a more frequent consumption of fried foods at baseline was associated with a higher risk of subsequently becoming overweight/obese during follow-up [40]. Therefore, high carbohydrate and/or wheat intake, and the deep-fried cooking method, might be the two determinant factors that contributed to the increased risk of obesity associated with the wheat staple dietary in men. By contrast, the wheat staple pattern for women was found to have no relationship with obesity. Compared with men, the pattern for women was more diverse in its food composition and comprised a combination of adverse and protective components. Apart from wheat flour (which women consumed much less of), soft drinks, especially sugar-sweetened drinks, were considered as adverse factors for obesity because of their low nutrients density and high-energy contents. Nevertheless, only coarse grains and fruits in the wheat staple pattern for women played a protective role against obesity. A high intake of coarse grains, which contain lower energy than rice and wheat flour, is recommended by The Chinese Dietary Guidelines because of their high contents of various vitamins and dietary fiber. Additionally, high intake of fruit also has a beneficial influence on the management of bodyweight. A recent study drew a similar conclusion that the greater baseline intake of fruit, but not vegetables or fiber, is associated with a lower risk of becoming overweight or obese in middle-aged and older women [41]. Therefore, under the comprehensive interaction of protective and adverse factors, the wheat staple pattern for women did not indicate a positive association with obesity.

Snacking patterns in our study shared similar food components for both genders, which were starchy roots/tubers, fruits, seeds/nuts, cakes/pastries, and soft drinks. In the stratified analyses, we found no association of the snacks pattern with general or abdominal obesity in both men and women. This non-significant relationship could be explained by the interaction of healthy and unhealthy food constituents in the snack pattern. On the one hand, the adverse food items, such as cakes/pastries and soft drinks were recognized to be associated with a higher risk of obesity because of their excess content of sugar and energy contents. On the other hand, fruits, seeds, and nuts, considered the healthy constituent in the pattern, would counteract the unhealthy effect on obesity. The linkage between snacking behavior and obesity is inconsistent among populations of different genders, age groups, regions, and income levels [16,42,43,44,45]. However, we believe that the diverse food composition of the snacks pattern in the different studies is the fundamental reason behind this discrepancy. Snack patterns mainly consist of high energy density and low-nutrient-content foods, which would increase the risk of obesity. Conversely, snacks patterns comprising nuts, seeds, and fruits will have a protective effect on weigh management. As a dietary ingredient, snacking behavior has been dramatically increasing in China since 2004, and the Chinese snacking composition is shifting from an unhealthy one to a healthy one [46]. Additionally, a marked transition from a traditional two or three meals per day toward meals combined with smacks is underway. Further research is needed to develop a better understanding of the relationship between snack patterns and obesity.

The prudent pattern in the present study was relatively healthy from the perspective of food composition for both genders. Men and women following this pattern consumed high amounts of coarse grains and fruits. In addition, dairy, tea, seafood, and processed meat were consumed appropriately by men and, particularly, by women. Obviously, apart from processed meats, the other food groups in prudent pattern will all play protective roles against obesity. The effects of coarse grains, fruits, and seafood have been discussed above. Dairy is indicated to play an important role in both prevention and treatment of obesity. The high dietary calcium present in dairy products plays a pivotal role in the regulation of energy metabolism, because high-calcium diets attenuate adipocyte lipid accretion and weight gain during the overconsumption of an energy-dense diet, and increase lipolysis and preserve thermogenesis during caloric restriction, which thereby markedly accelerates weight loss [47]. Additionally, epidemiological evidence and experimental studies have demonstrated that drinking tea is associated with a lower risk of general obesity and other metabolism syndromes. The beneficial effects of tea have been attributed to the presence of phenolic compounds that are powerful anti-oxidants and free iron scavengers [48]. Moreover, both men and women with high scores for the prudent pattern were more likely to have a healthy lifestyle. They had a higher education level, participated in more physical activity, smoked less (men) and did more housework. However, unlike other studies with a similar prudent pattern [49,50,51], we did not find a relationship between this pattern and obesity, which was probably because the sample size was not large enough to discover a statistically significant association. Therefore, further research with a larger sample size is necessary to examine the protective effects of the prudent dietary pattern in China.

Several limitations in this study should be mentioned. Firstly, the sample size was small. Although we detected statistically significant results for two patterns, further studies with larger sample sizes are needed to confirm this conclusion and for deeper exploration. Secondly, the conclusions cannot be extrapolated to the entire Chinese population, because the study participants are restricted to a middle-aged and elderly population in Shanghai. Thirdly, this study cannot show the resultant relationship between dietary patterns and obesity given the cross-sectional design. Finally, some subjective and arbitrary decisions, including determining the number of factors to retain, choosing the method of rotation of the initial factors, and labeling the dietary patterns, should be considered. In spite of these limitations, this is the first study to reveal the effect of gender difference on the association between dietary patterns and obesity in a middle-aged and elderly Chinese population. Furthermore, the use of the four periods of one-day recall method by a face-to-face interview and the adjustment for potential confounders, including age, education level, married status, occupation, smoking status, physical activity, and energy intake, ensured the validity and reliability of this study.

## 9. Conclusions

Our study identified four distinct dietary patterns for both genders of a middle-aged and elderly populations in Shanghai: the rice staple pattern, wheat staple pattern, snacks pattern, and prudent pattern. Our findings indicated that the rice staple pattern was associated positively with abdominal obesity in men, but was associated negatively with general obesity in women. A significant association between wheat staple pattern and men was observed, but not for women. Further prospective and larger sample size studies are needed to explore the gender difference in the association between dietary patterns and obesity.

## Figures and Tables

**Table 1:** Food groups in the factor analysis.

Food Groups	Examples of Food Items
Rice	Long-grained rice, round-grained rice, glutinous rice and products
Wheat	Wheat noodles, wheat buns and other wheat flour products
Deep fried wheat	Deep-fried dough sticks, deep-fried dough cakes
Instant noodles	Instant noodles
Coarse grains	Corn, oats, barley, sorghum foxtail millet
Starchy roots and tubers	Potatoes, taros, yams, lotus roots, sweet potatoes cassavas
Vegetables	Cabbage, spinach, tomatoes, cucumbers, zucchinis and products
Fruits	Fresh fruits and products
Pork	Pork
Poultry	Chickens, ducks, geese
Other livestock meats	Beef, lamb and other livestock meats
Organ meats	Livers, kidneys, large intestines, blood
Processed meats	Ham, luncheon meats, sausages, smoked meats, dried meats
Fresh water fish and seafood	Freshwater fish, saltwater fish, shrimp, crab and shellfish
Dairy	Animal-based milk, cheese, yogurt
Legumes	Soybeans, peas, mung beans, azuki beans and products
Eggs	Whole eggs, yolks, whites, preserved eggs
Seeds and nuts	Sesame seeds, peanuts, walnuts, almonds, cashews, pistachios
Fungi and algae	Mushroom, kelp and laver
Western fast food	Sandwiches, hamburgers, hotdogs, pizzas
Cakes and pastries	Cakes, cookies, moon cakes, pies and pastries
Candy and chocolates	Honey, sugar, candies, chocolate, Jelly
Soft drinks	Carbonated drinks, fruit juices and vegetable juices
Alcoholic beverages	Liquors, wine, beer vodka, cocktails, whiskey
Tea	Black tea, green tea, oolong tea

**Table 2:** Characteristics of the study participants.

Variable	Total	Men	Women	*p* Value
No. (%)	2046	968 (47.3%)	1078 (52.7%)	
Age in years (%)	60.1 ± 10.8	60.0 ± 10.7	60.2 ± 10.9	
45–59	1031	477 (49.3)	554 (51.4)	0.340
60–	1015	491 (50.7)	524 (48.6)	
BMI (%)				
under weight	72 (3.5)	30 (3.0)	42 (3.9)	0.017
normal	1039 (50.8)	476 (49.2)	563 (52.2)	
overweight	745 (36.4)	384 (39.7)	361 (33.5)	
general obesity	190 (9.3)	78 (8.1)	112 (10.4)	
abdominal obesity (%)	972 (47.5)	512 (52.9)	460 (42.7)	<0.001
education level (%)				
Lower	1432 (70.0)	646 (66.7)	786 (72.9)	0.002
Higher	614 (30.0)	322 (33.3)	292 (27.1)	
marital status2 (%)				
married	1864 (91.1)	912 (94.2)	952 (88.3)	<0.001
other marital status	182 (8.9)	56 (5.8)	126 (11.7)	
smoking status (%)				
never	1485 (72.6)	415 (42.9)	1070 (99.3)	<0.001
former	52 (2.5)	52 (5.4)	0 (0.0)	
current	509 (24.9)	501 (51.8)	8 (0.7)	
occupation (%)				
retired	1228 (63)	572 (59.1)	716 (66.4)	<0.001
others	758 (37)	396 (40.9)	362 (33.6)	
physical activity (%)				
light	1105 (54)	550 (56.8)	555 (51.5)	0.054
moderate	370 (18,1)	164 (16.9)	206 (19.1)	
vigorous	571 (27.9)	254 (26.2)	317 (29.4)	
housework status (%)				
yes	1654 (80.8)	657 (67.9)	997 (92.5)	<0.001
no	392 (19.2)	311 (32.1)	81 (7.5)	

**Table 3:** Factor-loading matrix for dietary patterns among 2046 Shanghai residents aged over 45 years.

Food Groups	Men (*n* = 968)				Women (*n* = 1078)			
	Rice Staple	Wheat Staple	Snacks	Prudent	Rice Staple	Wheat Staple	Snacks	Prudent
Rice	0.413	−0.503	−0.462		0.415	−0.565		
Wheat		0.619				0.575		
Deep fried wheat		0.213						
Instant noodles	−0.221	0.213						
Coarse grains				0.966		0.278		0.929
Starchy roots and tubers	0.295	−0.439	0.352				0.428	
Vegetables	0.580		−0.370		0.684			
Fruits			0.277	0.966		0.280	0.464	0.928
Pork	0.244		−0.216	−0.202	0.267		−0.279	
Poultry	0.571				0.319			
Other livestock meats			-					
Organ meats	0.226							
Processed meats	0.466					−0.355		0.274
Fresh water fish and seafood		−0.447			0.286			0.203
Dairy		0.383		0.317		0.591		
Legumes		0.370				0.264		
Eggs			−0.446		0.464			
Seeds and nuts	0.288					0.201	0.669	
Fungi and algae	0.312				0.474	0.200		
Western fast food								
Cakes and pastries			0.433				0.541	
Candy and chocolates								
Soft drinks			0.407			0.363		
Alcoholic beverages			0.306					
Tea		0.344	0.201	0.220		0.236		

**Table 4:** Characteristics of participants by quartiles (Q) of dietary pattern scores

	Rice Staple			Wheat Staple			Snacks			Prudent		
	Q1 (Low)	Q4 (High)	*p*	Q1 (Low)	Q4 (High)	*p*	Q1 (Low)	Q4 (High)	*p*	Q1 (Low)	Q4(High)	*p*
**Men**												
No.	242	242		242	242		242	242		242	242	
Age in year	61.0 ± 11.0	58.8 ± 10.8	0.024	59.4 ± 9.9	60.1 ± 11.0	0.460	58.5 ± 9.8	61.0 ± 11.7	0.011	58.1 ± 9.8	62.1 ± 11.6	<0.001
BMI (kg/m^2^)	23.5 ± 3.2	24.1 ± 3.4	0.059	23.7 ± 3.5	23.9 ± 2.8	0.495	23.8 ± 3.3	23.6 ± 3.1	0.471	23.9 ± 3.3	23.8 ± 3.3	0.510
WC (cm)	84.5 ± 9.2	86.4 ± 8.5	0.021	84.5 ± 8.9	86.6 ± 8.6	0.010	85.1 ± 8.9	86.0 ± 9.5	0.292	86.1 ± 9.9	86.0 ± 9.5	0.950
General obesity (%)	16 (6.6)	30 (12.4)	0.030	23 (9.5)	13 (5.4)	0.083	19 (7.9)	14 (5.8)	0.367	18 (7.4)	23 (9.5)	0.414
Abdominal obesity (%)	103 (42.6)	138 (57.0)	0.001	105 (43.4)	142 (58.7)	0.001	122 (50.4)	125 (51.7)	0.785	126 (52.1)	133 (55.0)	0.524
Education (high,%)	70 (28.9)	81 (33.5)	0.280	41 (16.9)	131 (54.1)	<0.001	57 (23.6)	101 (41.7)	<0.001	47 (19.4)	121 (50)	<0.001
Married (%)	210 (89.4)	225 (95.7)	0.008	222 (94.5)	226 (95.0)	0.812	225 (95.7)	219 (93.6)	0.299	217 (91.9)	229 (95.8)	0.078
Smoking status (%)												
Never	99 (40.9)	111 (45.9)	0.355	110 (45.5)	102 (42.1)	0.764	111 (45.9)	104 (43.0)	0.393	81 (33.5)	121 (50.0)	<0.001
Former	13 (5.4)	8 (3.3)		15 (6.2)	16 (6.6)		8 (3.3)	14 (5.8)		9 (3.7)	17 (7.0)	
Current	130 (53.7)	123 (50.8)		117 (48.3)	124 (51.2)		123 (50.8)	124 (51.2)		152 (62.8)	104 (43.0)	
Occupation (retired, %)	154 (63.6)	126 (52.1)	0.010	120 (49.6)	158 (65.3)	<0.001	130 (53.7)	154 (63.6)	0.027	125 (51.7)	176 (72.7)	<0.001
Physical activity												
Light	146 (60.3)	131 (54.1)	0.143	158 (65.3)	108 (44.6)	<0.001	154 (63.6)	122 (50.4)	0.002	180 (74.4)	104 (43.0)	<0.001
Moderate	43 (17.8)	39 (16.1)		46 (19.0)	41 (16.9)		45 (18.6)	44 (18.2)		29 (12.0)	54 (22.3)	
Vigorous	53 (21.9)	72 (29.8)		38 (15.7)	93 (38.4)		43 (17.8)	76 (31.4)		33 (13.6)	84 (34.7)	
Doing housework (%)	157 (64.9)	170 (70.2)	0.207	163 (67.4)	166 (68.6)	0.770	164 (67.8)	168 (69.4)	0.695	139 (57.4)	182 (75.2)	<0.001
Total energy intake (Kcal/day)	1580.30 ± 589.63	1661.78 ± 557.47	0.119	1583.66 ± 614.50	1646.20 ± 534.41	0.233	1611.21 ± 602.33	1642.67 ± 588.04	0.561	1574.59 ± 614.65	1639.56 ± 518.05	0.209
**Women**												
No.	269	269		269	269		269	269		269	269	
Age in year	63.0 ± 12.4	58.9 ± 9.5	<0.001	59.6 ± 10.7	61.0 ± 10.9	0.123	58.2 ± 9.6	61.2 ± 11.5	0.001	60.4 ± 11.1	59.6 ± 10.9	0.397
BMI (kg/m^2^)	23.6 ± 3.8	23.8 ± 3.1	0.698	23.9 ± 3.6	23.4 ± 3.0	0.056	23.8 ± 3.5	24.1 ± 3.4	0.339	23.7 ± 3.4	23.7 ± 3.5	0.858
WC (cm)	80.7 ± 9.1	79.6 ± 8.4	0.149	80.1 ± 8.4	79.2 ± 8.7	0.246	79.8 ± 8.6	80.2 ± 8.7	0.588	80.5 ± 9.1	78.9 ± 8.7	0.042
General obesity (%)	38 (14.1)	24 (8.9)	0.059	26 (9.7)	20 (7.4)	0.355	33 (12.3)	30 (11.2)	0.688	32 (11.9)	25 (9.3)	0.327
Abdominal obesity (%)	132 (49.1)	105 (39.9)	0.019	116 (43.1)	112 (41.6)	0.727	111 (41.3)	122 (45.4)	0.339	120 (44.6)	106 (39.4)	0.221
Education (high, %)	204 (75.8)	196 (72.9)	0.430	25 (9.3)	133 (49.4)	<0.001	60 (22.3)	83 (30.9)	0.025	36 (13.4)	106 (39.4)	<0.001
Married (%)	211 (79.0)	234 (91.1)	<0.001	224 (86.8)	231 (88.2)	0.643	236 (91.5)	222 (84.4)	0.013	225 (86.9)	230 (87.1)	0.933
Occupation (retired, %)	186 (69.1)	183 (68.0)	0.781	140 (52.0)	207 (77.0)	<0.001	164 (61.0)	191 (71.0)	0.014	162 (60.2)	208 (77.3)	<0.001
Physical activity												
light	149 (55.4)	125 (46.5)	0.054	175 (65.1)	100 (37.2)	<0.001	144 (53.5)	127 (47.2)	0.305	160 (59.5)	128 (47.6)	<0.016
moderate	41 (15.2)	60 (22.3)		52 (19.3)	51 (19.0)		52 (19.3)	55 (20.4)		43 (16.0)	49 (18.2)	
vigorous	79 (29.4)	84 (31.2)		42 (15.6)	118 (43.9)		73 (27.1)	87 (32.3)		66 (24.5)	92 (34.2)	
Doing housework (%)	241 (89.6)	255 (94.8)	0.024	249 (92.6)	249 ((92.6)	1.000	256 (95.2)	246 (91.4)	0.084	248 (92.2)	249 (92.6)	0.871
Total energy intake (Kcal/day)	1510.02 ± 672.77	1634.41 ± 744.62	0.043	1540.94 ± 656.87	1627.23 ± 690.34	0.138	1611.21 ± 706.96	1705.35 ± 866.22	0.168	1603.25 ± 736.61	1631.64 ± 606.10	0.626

**Table 5:** Association of dietary patterns with general obesity and abdominal obesity across quartiles of dietary pattern scores.

Gender	Dietary Patterns	General Obesity			Abdominal Obesity		
		Q1	Q4	*p*	Q1	Q4	*p*
**Men**	**Rice staple**						
	Model 1.	1	1.873 (1.062, 3.441)	0.035	1	1.383 (1.153, 1.668)	0.001
	Model 2.	1	1.836 (1.031, 3.399)	0.044	1	1.358 (1.132, 1.639)	0.001
	Model 3.	1	1.800 (0.998, 3.226)	0.054	1	1.358 (1.132, 1.639)	0.001
	**Wheat staple**						
	Model 1.	1	0.566 (0.285, 1.074)	0.089	1	1.349 (1.131, 1.619)	0.001
	Model 2.	1	0.631 (0.297, 1.290)	0.216	1	1.331 (1.094, 1.627)	0.005
	Model 3.	1	0.621 (0.294, 1.260)	0.196	1	1.331 (1.094, 1.627)	0.005
	**Snacks**						
	Model 1.	1	0.830 (0.519, 1.169)	0.362	1	1.006 (0.844, 1.201)	0.942
	Model 2.	1	0.891 (0.545, 1.083)	0.587	1	1.022 (0.854, 1.224)	0.809
	Model 3.	1	0.887 (0.541, 1.078)	0.574	1	1.023 (0.856, 1.225)	0.797
	**Prudent**						
	Model 1.	1	1.236 (0.896, 1.270)	0.362	1	1.031 (0.872, 1.221)	0.719
	Model 2.	1	1.022 (0.939, 1.245)	0.799	1	0.996 (0.816, 1.216)	0.967
	Model 3.	1	1.022 (0.939, 1.245)	0.770	1	0.997 (0.817, 1.217)	0.978
**Women**	**Rice staple**						
	Model 1.	1	0.863 (0.613, 1.136)	0.076	1	0.826 (0.678, 1.001)	0.053
	Model 2.	1	0.749 (0.626, 0.912)	0.038	1	0.839 (0.687, 1.018)	0.079
	Model 3.	1	0.745 (0.673, 0.807)	0.031	1	0.832 (0.681, 1.011)	0.067
	**Wheat staple**						
	Model 1.	1	0.857 (0.583, 1.157)	0.375	1	0.948 (0.781, 1.150)	0.586
	Model 2.	1	1.008 (0.706, 2.397)	0.950	1	1.000 (0.802, 1.244)	0.997
	Model 3.	1	1.014 (0.694, 1.028)	0.903	1	1.000 (0.802, 1.244)	0.997
	**Snacks**						
	Model 1.	1	0.941 (0.697, 1.201)	0.660	1	1.066 (0.883, 1.291)	0.507
	Model 2.	1	0.976 (0.711, 1.259)	0.865	1	1.100 (0.905, 1.339)	0.337
	Model 3.	1	0.972 (0.849, 1.080)	0.823	1	1.100 (0.904, 1.340)	0.340
	**Prudent**						
	Model 1.	1	0.863 (0.613, 1.136)	0.345	1	0.916 (0.751, 1.113)	0.380
	Model 2.	1	0.915 (0.672, 1.085)	0.544	1	0.961 (0.777, 1.183)	0.710
	Model 3.	1	0.915 (0.784, 1.085)	0.545	1	0.959 (0.776, 1.179)	0.692

Model 1: adjusted for age (continuous). Model 2: model 1 additionally adjusted for education level (lower, higher), marital status (married, others), smoking status (never, former, current but not included in women), occupation (retired, others), physical activity (light, moderate and vigorous), and housework status (yes/no). Model 3: model 2 further adjusted for total energy intake (continuous).

## Appendix A

**Table A1:** Absolute amount of food groups by quartiles (Q) of dietary pattern scores.

	Rice Staple			Wheat Staple			Snacks			Prudent		
	Q1 (Low)	Q4 (High)	*p*	Q1 (Low)	Q4 (High)	*p*	Q1 (Low)	Q4 (High)	*p*	Q1 (Low)	Q4 (High)	*p*
Men/Women (g)												
Rice	217.4/194.8	390.9/368.1	<0.001/<0.001	523.0/393.9	235.3/171.1	<0.001/<0.001	497.9/285.5	250.0/270.4	<1.001/0.274	392.2/321.1	307.1/242.8	<0.001/<0.001
Wheat	72.0/81.3	70.1/51.8	0.876/<0.001	15.6/12.0	162.3/119.9	<0.001/<0.001	63.1/85.2	83.9/47.8	<0.001/<0.001	69.8/62.1	82.0/65.4	0.012/0.660
Deep fried wheat	2.0/1.6	3.0/1.4	0.475/0.800	0.32/1.8	5.5/1.3	<0.001/0.461	1.9/2.4	2.1/1.2	0.823/0.135	1.3/0.2	3.6/2.8	0.068/0.001
Instant noodles	2.3/0.8	0.0/0.3	0.01/0.347	0.8/0.0	13.2/1.6	<0.001/0.176	0.0/0.0	0.8/0.9	0.062/0.051	0.0/1.5	1.0/0.0	0.079/0.202
Coarse grains	128.5/147.4	148.8/185.0	0.151/0.007	122.6/101.2	158.0/251.6	0.016/<0.001	129.9/156.2	137.7/194.9	0.601/0.005	5.1/23.7	323.7/374.0	<0.001/<0.001
Starchy roots and tubers	8.9/17.0	38.5/24.2	<0.001/0.043	45.7/30.1	9.7/19.9	<0.001/<0.05	12.1/5.8	43.3/44.9	<0.001/<0.001	13.8/17.9	27.5/29.4	0.014/0.023
Vegetables	176.8/152.9	405.8/417.4	<0.001/<0.001	305.3/280.8	254.7/242.4	0.004/0.016	404.7/264.2	217.8/267.4	<0.001/0.803	291.0/263.4	290.1/261.4	0.961/0.893
Fruits	128.9/149.7	150.8/187.2	0.124/0.007	123.6/102.7	159.7/254.8	0.015/<0.001	131.6/157.8	239.2/377.9	0.006/0.003	5.2/25.2	326.4/376.1	<0.001/<0.001
Pork	57.0/45.2	112.7/94.7	<0.001/<0.001	86.6/54.5	85.7/74.1	0.897/<0.001	120.1/108.6	68.4/50.3	<0.001/<0.001	113.5/75.9	66.1/57.1	<0.001/<0.001
Poultry	1.5/5.1	38.9/24.5	<0.001/<0.001	8.4/11.5	7.5/15.8	<0.001/0.107	11.1/29.6	18.7/32.1	0.022/<0.188	11.5/13.8	20.4/12.7	0.008/0.699
Other livestock meats	3.6/1.6	9.6/8.5	0.009/<0.001	1.4/2.0	14.0/5.7	<0.001/0.003	7.0/7.1	6.5/2.5	0.805/0.002	3.0/3.0	8.3/5.5	0.011/0.043
Organ meats	0.4/1.4	5.2/1.8	<0.001/0.593	4.0/2.3	0.7/0.8	0.008/0.034	4.4/0.2	6.5/2.9	<0.643/<0.001	3.4/0.3	1.1/2.4	0.039/0.003
Processed meats	0.6/7.1	17.4/4.8	<0.001/0.164	8.8/12.3	7.5/1.7	0.576/<0.001	6.9/1.9	10.2/10.3	0.157/<0.001	8.8/0.5	5.7/11.9	0.128/<0.001
Fresh water fish and seafood	85.5/33.8	87.7/88.0	0.895/<0.001	133.1/68.4	51.4/67.8	<0.001/0.937	78.6/81.9	99.0/58.2	0.115/0.001	52.2/43.8	112.2/84.8	<0.001/<0.001
Dairy	49.7/48.8	38.4/65.2	<0.119/0.035	6.2/4.2	93.4/139.1	<0.001/<0.001	22.9/48.1	69.7/71.0	<0.001/0.003	8.6/46.8	83.6/69.5	<0.001/0.003
Legumes	14.2/17.6	34.8/27.8	<0.001/0.001	13.3/12.3	37.9/31.5	<0.001/<0.001	25.3/16.2	26.9/27.3	0.645/<0.001	26.2/20.4	28.1/23.7	<0.001/0.290
Eggs	35.3/18.7	46.7/69.6	0.012/<0.001	38.9/39.2	37.1/39.0	0.665/0.959	76.1/55.0	24.0/32.1	<0.001/<0.001	30.9/37.0	41.2/41.9	0.013/0.230
Seeds and nuts	1.2/5.5	17.3/12.9	<0.001/<0.001	8.3/4.3	6.4/16.0	0.299/<0.001	5.9/0.8	11.1/26.7	0.01/<0.001	7.9/16.0	8.3/6.8	0.837/0.002
Fungi and algae	3.0/3.1	23.4/25.2	<0.001/<0.001	5.7/5.0	15.7/17.7	<0.001/<0.001	12.3/8.8	11.7/16.2	0.808/0.001	6.5/11.8	16.2/11.1	<0.001/0.751
Western fast food	0.0/0.5	0.6/0.0	0.318/0.318	0.0/0.6	0.6/0.0	0.318/<0.318	0.0/0.0	0.6/0.7	0.318/0.206	0.6/0.0	0.0/0.0	0.318/-
Cakes and pastries	3.4/12.7	11.4/4.1	<0.001/<0.001	5.5/4.0	10.6/13.2	0.005/<0.001	1.0/0.6	20.3/21.6	<0.001/0.001<	4.3/7.0	10.4/7.5	<0.001/0.727
Candy and chocolates	5.9/7.6	1.9/1.1	0.005/0.030	8.2/2.6	1.3/5.3	0.032/0.350	1.6/7.7	6.8/3.9	0.085/0.078	0.5/5.3	8.1/2.8	0.014/0.319
Soft drinks	11.1/28.5	78.0/63.6	<0.001/0.044	35.4/8.6	36.6/119.7	0.925/<0.001	3.9/34.4	111.1/48.8	<0.001/0.261	39.2/35.8	41.4/55.3	0.880/0.149
Alcoholic beverages	26.8/0.1	34.3/2.6	0.485/0.100	23.9/1.3	26.9/1.6	0.724/0.876	2.2/0.1	74.3/2.5	<0.001/0.101	58.4/0.8	16.2/0.7	<0.001/0.986
Tea	271.1/270.0	380.1/288.4	0.024/0.648	102.1/142.2	666.1/461.4	<0.001/<0.001	134.1/419.6	506.2/294.9	<0.001/0.011	162.7/341.1	507.7/325.8	<0.001/0.756
